# *Onchocerca volvulus* infection in Tihama region - west of Yemen: Continuing transmission in ivermectin-targeted endemic foci and unveiled endemicity in districts with previously unknown status

**DOI:** 10.1371/journal.pntd.0006329

**Published:** 2018-03-05

**Authors:** Mohammed A. K. Mahdy, Rashad Abdul-Ghani, Thaker A. A. Abdulrahman, Samira M. A. Al-Eryani, Abdulsalam M. Al-Mekhlafi, Sami A. A. Alhaidari, Ahmed A. Azazy

**Affiliations:** 1 Tropical Disease Research Center, University of Science and Technology, Sana’a, Yemen; 2 Department of Parasitology, Faculty of Medicine and Health Sciences, Sana’a University, Sana’a, Yemen; 3 Health Services Sector, the Charitable Society for Social Welfare, Sana’a, Yemen; 4 National Schistosomiasis and Parasites Control Program, Ministry of Public Health and Population, Sana’a, Yemen; 5 Department of Laboratory Medicine, Faculty of Applied Medical Sciences, Al-Baha University, Al-Baha, KSA; RTI International, UNITED STATES

## Abstract

**Background:**

Onchocerciasis in Yemen is one of the most neglected diseases, where baseline estimates of onchocerciasis and monitoring of the impact of ivermectin regularly administered to the affected individuals on its transmission are lacking. Therefore, this study aimed to determine the anti-*Ov*16 IgG4 seroprevalence among local communities of Hodeidah and Al-Mahwit governorates of Tihama region. The factors possibly associated with previous exposure to infection were also studied.

**Methodology/Principal findings:**

This cross-sectional study was conducted in two ivermectin-targeted districts endemic for onchocerciasis in Hodeidah and Al-Mahwit and two untargeted districts with unknown previous endemicity in Hodeidah between February and July 2017. For 508 residents sampled by a multi-stage random approach, data were collected and blood specimens were screened for anti-*Ov*16 IgG4 using the SD BIOLINE Onchocerciasis IgG4 rapid tests. The study revealed an overall anti-*Ov*16 IgG4 rate of 18.5% (94/508) in all surveyed districts, with 10.2% (12/118) of children aged ≤10 years being seropositive. Moreover, rates of 8.0% (4/50) and 6.1% (4/66) were found in districts not officially listed as endemic for the disease. Multivariable analysis confirmed the age of more than ten years and residing within a large family as the independent predictors of exposure to infection.

**Conclusions/Significance:**

Onchocerciasis transmission is still ongoing as supported by the higher anti-*Ov*16 IgG4 seroprevalence rate among children aged ≤10 years compared to that (<0.1%) previously set by the World Health Organization as a serologic criterion for transmission interruption. Further large-scale studies combining serologic and entomologic criteria are recommended for the mapping of *O*. *volvulus* in human and blackfly populations in endemic foci and their neighboring areas of uncertain endemicity. In addition, ivermectin distribution, coverage and impact on disease transmission need to be continually assessed.

## Author summary

Onchocerciasis is endemic in certain foci in the western governorates of Yemen. Monitoring the impact of the regular ivermectin administration to affected individuals on the transmission status and providing baseline onchocerciasis estimates in endemic areas are crucial for planning effective elimination strategies. We found that the disease transmission is still ongoing in Hodeidah and Al-Mahwit governorates of Tihama region as indicated by the anti-*Ov*16 IgG4 seropositivity among children aged ≤10 years. In Bani Sa'ad, where affected individuals had been regularly targeted with ivermectin over the last 15 years, we found that the anti-*Ov*16 IgG4 seroprevalence rate was significantly lower among children aged ≤10 years (9.1%; 5/55) compared to those >10 years (24.5%; 37/151), reflecting a possible decline in disease transmission. We also revealed onchocerciasis transmission in districts with unknown previous endemicity for the first time, with rates of 8.0% and 6.1% being found in Al Marawi'ah and As Sukhnah districts of Hodeidah. Large-scale surveys are recommended for mapping of *O*. *volvulus* in human and blackfly populations in endemic foci and neighboring untargeted areas of uncertain endemicity as a forward step towards the elimination of the disease from the country.

## Introduction

Onchocerciasis is a neglected tropical disease of the skin and eyes caused by the filarial nematode *Onchocerca volvulus* and transmitted by the bites of infected *Simulium* blackflies. It is endemic in 31 countries in sub-Saharan Africa and in some foci in Latin America and Yemen, with approximately 187 million people being exposed to potential transmission [1, 2]. In addition, over a million disability-adjusted life years have been recently estimated to be lost due to onchocerciasis [3]. Promising strides towards the control and elimination of the disease have been made since the introduction and donation of the microfilaricide ivermectin (Mectizan) through Mectizan Donation Program (MDP) in the late 1980s [4–7]. Ivermectin administration at intervals interrupts transmission and incidence of new infections with *O*. *volvulus* in endemic foci in the long run [8, 9]. Effective efforts through mass drug administration (MDA) campaigns at repeated rounds undertaken by control programs have led to the successful elimination of the disease in four countries in Latin America as certified by the World Health Organization (WHO) between 2013 and 2016; namely, Colombia, Ecuador, Mexico and Guatemala [10].

Yemen is the only country endemic for onchocerciasis in Asia, where the disease mainly affects the rural communities residing near the flowing streams of main seasonal watercourses (locally referred to as wadis) in western governorates [11, 12]. Clinically, onchocerciasis in Yemen is a unique form of localized, hyper-reactive onchodermatitis referred to as "sowda" [13], which is difficult to diagnose in the laboratory by skin snip examination as a result of the scarcity of microfilariae [14, 15]. Although the epidemiology of onchocerciasis in the country lacks clear mapping and national burden estimates, its focal endemicity has been documented in 33 districts of eight governorates; namely, Taiz, Ibb, Hodeidah, Dhamar, Raymah, Al-Mahwit, Sana’a and Hajjah [12].

In the early 1990s, ivermectin donated by the MDP was first distributed for treating the clinical manifestations of sowda in Wadi Al-Ghail, Taiz [16], where onchocerciasis had been reported to be endemic by Büttner et al. [11]. Its use at three-month intervals was then recommended as a control strategy, desirably through national campaigns [16]. Ivermectin has then been distributed to patients in a few affected communities, mainly through the National Leprosy Elimination Program in Taiz and the Charitable Society for Social Welfare (CSSW), a non-governmental organization (NGO) committed to Mectizan distribution to the affected populations since 2000. Several campaigns have been implemented in endemic areas following the approval of donating Yemen 91,000 Mectizan treatments on a quarterly basis by the Mectizan Expert Committee of the MDP [6].

The political crisis and war in the country since the Arab Spring revolutions in the region in 2011 have dashed the hope raised by the development of a national action plan in 2010 to eliminate the disease by 2015 [17]. The major mainstays adopted as part of the onchocerciasis elimination plan involve a combination of MDA with ivermectin to at-risk populations together with consolidating the clinic-based management of infected cases. In addition, the plan involves vector control and strengthening surveillance, including serologic and entomologic surveys (Ministry of Health and Population, personal communication, 2018). However, the current situation led to a number of challenges to the implementation of the elimination plan, including the insecurity, financial and logistic restrains besides the humanitarian priorities. In January 2016, however, the first MDA with ivermectin was implemented in Hodeidah and Al-Mahwit, targeting over 162,000 children and adults [18]. Although the disease is of focal nature and its baseline mapping in the targeted governorates is lacking, an ivermectin coverage rate of 94.8% has been reported in four targeted districts in the two governorates of Tihama region (Ministry of Health and Population, personal communication, 2018). It is worth mentioning, and to the best of our knowledge, that there are no published studies on the serostatus of onchocerciasis in the targeted areas of the country. Defining areas to be targeted by MDA with ivermectin and post-MDA surveys are key components to the success of the proposed elimination plan. This, in turn, highlights the importance of the present pilot study in providing a zoomed image to a part of the epidemiologic scene from its serologic attribute.

Serologic markers are now widely used to determine the recent exposure to infection with *O*. *volvulus* because of the possibility of their early detection in infection before skin snips become positive. In this regard, immunoglobulin G4 (IgG4) response to the *Ov*16 antigen expressed by the third (L3) and fourth (L4) larval stages of the parasite is the most specific marker of recent infection [19], confirming ongoing disease transmission. This marker is highly sensitive and provides evidence for recent transmission when detected among young children. Accordingly, the negativity of anti-*Ov*16 IgG4 has been recently used to confirm the interruption of disease transmission in foci following extensive rounds of MDA or community-directed treatment with ivermectin (CDTI) campaigns in a number of countries in Latin America and Africa [20–24].

When tested against skin microfilaria status, a lateral flow rapid diagnostic test (RDT) for detecting anti-*Ov*16 IgG4 antibodies against the parasite showed sensitivity and specificity levels of 98.0% compared to levels of 94.0% and 96.0%, respectively, for enzyme-linked immunosorbent assay (ELISA) [25]. This, in turn, makes the use of RDTs for detecting anti-*Ov*16 in sera of children in endemic settings a useful and cost-effective tool for the long-term monitoring of disease transmission in the community following MDA campaigns [26, 27]. In 2014, the SD BIOLINE Onchocerciasis IgG4 RDT was launched as a surveillance tool for identifying exposure to infection by detecting anti-*Ov*16 IgG4 [28]. It is noteworthy that the quality of such RDTs during field use has been successfully ensured with the use of recombinant human anti-*Ov*16 IgG4 antibody-based positive controls [29].

In line with the efforts to eliminate onchocerciasis from the country, there is a need to evaluate the status of its ongoing transmission after ivermectin distribution campaigns in endemic foci. Therefore, the present study aimed to determine anti-*Ov*16 IgG4 serostatus among rural residents in ivermectin-targeted endemic areas and neighboring untargeted areas with unknown endemicity in Hodeidah and Al-Mahwit governorates of Tihama region, west of Yemen. The factors possibly associated with previous exposure to *O*. *volvulus* infection were also studied.

## Methods

### Study design and setting

**Fig 1:**
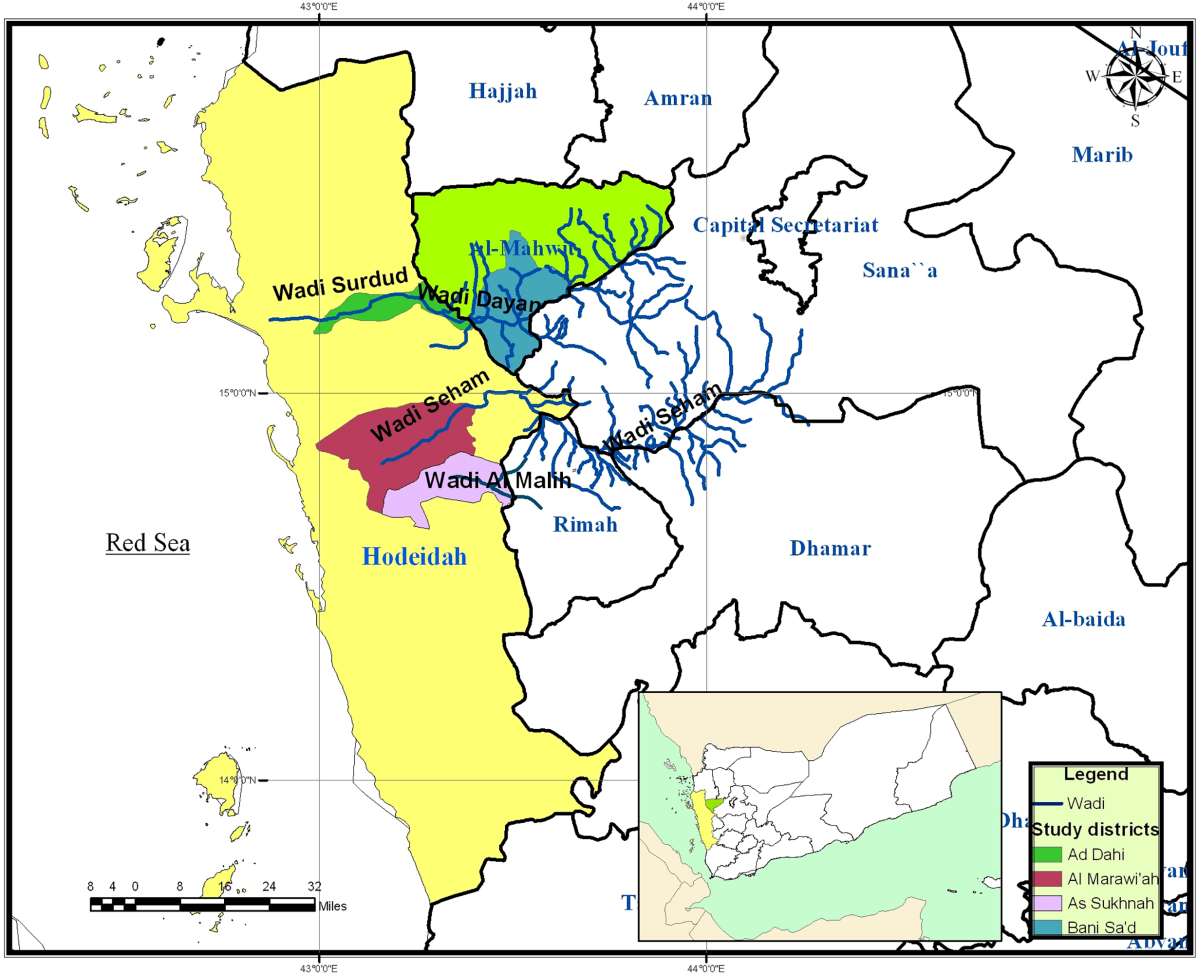
Map of the study area.

This cross-sectional study (S1 Checklist) was conducted in four districts in Hodeidah and Al-Mahwit in the period from February to July 2017. Hodeidah is located on the Red Sea at the coordinates of 14°48' N and 42°75' E, whereas Al-Mahwit is bordering Hodeidah and located at the coordinates of 15°28' N and 43°32' E (Fig 1). Both governorates are characterized by the presence of fast-flowing seasonal streams and perennial watercourses (wadis), where Wadi Surdud is the most famous one traversing the two governorates to drain into the Red Sea. It is well-known that the people of rural areas residing alongside these watercourses are mainly engaged in agricultural activities.

Of the four districts surveyed during the present study, two are endemic for onchocerciasis; namely, Ad Dahi alongside Wadi Surdud and its tributaries in Hodeidah and Bani Sa'ad alongside Wadi Dayan and its tributaries in Al-Mahwit, where breeding sites of the vector exist. The first CDTI campaign in Tihama region was implemented in both districts in 2016 (CSSW, personal communication, 2017). Moreover, ivermectin distribution campaigns targeting symptomatic patients have been conducted three times a year since 2002 in Bani Sa'ad. Meanwhile, Al Marawi'ah and As Sukhnah districts of Hodeidah in the vicinity of the surveyed endemic districts, which are not listed as onchocerciasis-endemic districts (Ministry of Public Health and Population, personal communication, 2017), were included in the study. Al Marawi'ah is traversed by Wadi Siham and its tributaries, while As Sukhnah is traversed by Wadi Malih, which may be potential breeding sites for the vector.

### Sample size and sampling strategy

In accordance with the criteria set by the WHO for the determination of sample size in health studies [30], a minimum sample size of 384 was calculated at an expected onchocerciasis prevalence of 50.0% (due to the lack of prevalence data in the country), a confidence level of 95.0% and an accepted margin of error of 5.0%. Yet, 392 individuals were recruited from the surveyed onchocerciasis-endemic districts.

To avoid the effect that might be introduced as a result of the heterogeneity in infection prevalence and the sparse distribution of rural communities in the study areas, multi-stage sampling was adopted to obtain the best representative sample, where endemic districts and sub-districts of the studied governorates were considered as the clusters. In the first stage, Ad Dahi and Bani Sa'ad were randomly selected from a list of endemic districts in Hodeidah and Al-Mahwit, respectively. In the second stage, two (Upper Grabeh and Lower Grabeh) and four (Al Wahaweh, Bani Ali, Gaaferat Alh and Utmah) sub-districts were randomly selected from Ad Dahi and Bani Sa'ad, respectively. It is to be noted that cluster sampling might not always have a relationship to streams and possible breeding sites, which could yield a somewhat biased sample close to breeding sites to ensure negativity at the source of the infection. Households were then randomly selected from each sub-district, and all family members were invited to participate, ensuring the proportionality of the sample size of each sub-district to its population size. In addition to the individuals sampled from onchocerciasis-endemic districts, 116 individuals were randomly selected from Al Marawi'ah and As Sukhnah districts with unknown previous endemicity, totaling the sample size to 508.

### Data collection and blood screening for anti-*Ov*16 IgG4

Data on the district of residence, gender, age, clinical signs of onchocerciasis, source of drinking water, durables of households and history of ivermectin intake were collected using a pre-designed questionnaire. Presence of nodules and the subjective reporting of itching that usually disturbs sleeping or interferes with working capacity were recorded according to the guidelines of the WHO [31]. However, it was almost impossible to observe the nodules in body parts that are considered to be private by the participants, particularly women. Finger-prick blood was screened for anti-*Ov*16 IgG4 using the SD BIOLINE Onchocerciasis IgG4 RDT (Standard Diagnostics, Inc., Gyeonggi-do, Republic of Korea) according to the manufacturer’s instructions. Negative and three concentrations of positive controls (high, middle and low concentrations of anti-*Ov*16 IgG4), supplied by PATH (www.path.org), were used to ensure the quality of each lot of RDTs at the points of testing in the field prior to blood screening.

### Data analysis

Data were analyzed using IBM SPSS Statistics for Windows, version 23.0 (IBM Corp., Armonk, NY, USA). The socioeconomic status (SES) was determined using the principal component analysis (PCA) of durables owned by households [32]. The constructed PCA-based scores of households were divided into five wealth quintiles and three SES categories, where households’ residents with the lowest 40%, the middle 20% and the highest 40% of household wealth quintiles were classified as being of low, middle and high SES, respectively [32].

Associations or differences between categorical variables were tested using Pearson’s chi-square test in bivariate analysis. The crude odds ratios (ORs) and the associated 95% confidence intervals (CIs) of the proportion of seropositive individuals were also calculated to measure the strength of association between each independent categorical variable and the anti-*Ov*16 IgG4 seropositivity status. Multivariable analysis using logistic regression was performed to determine the adjusted ORs with their associated 95% CIs so as to identify the independent predictors of anti-*Ov*16 IgG4 seropositivity. *P* values of < 0.05 were considered statistically significant.

### Ethical statement

The study protocol was reviewed and approved by the Ethics Committee of the Faculty of Medicine and Health Sciences, University of Science and Technology, Sana’a, Yemen (Ref. 2016/14). Participation in the study was on a voluntary basis after explaining its purpose to the heads of households and participants. In this respect, written informed consent was obtained from adults and the heads of households before recruiting their children.

## Results

Of the 508 individuals screened for anti-*Ov*16 IgG4, the majority were females (56.7%), children aged <11 years (23.2%), not educated (55.9%) and not working (67.5%). The median age of participants was 20 years (interquartile range: 11–38). The proportion of participants with a history of receiving ivermectin was 42.7% (217/508). Regarding the SES, individuals belonging to the high and low SES were equally distributed, representing 39.8% (156/392) of the study population each. However, 20.4% (80/392) of the participants were of the middle SES. Of the study subjects, 48.0% were living in huts or houses composed of one room (Table 1).

**Table 1:** Characteristics of study participants (N = 508).

Characteristic	*n* (%)
**District of residence**	
Bani Sa’ad	206 (40.6)
Ad Dahi	186 (36.6)
Al Marawi’ah	50 (9.8)
As Sukhnah	66 (13.0)
**Gender**	
Female	288 (56.7)
Male	220 (43.3)
**Age** (years)	
≤10	118 (23.2)
>10	390 (76.8)
**Household’s size** (members)*	
≤5	56 (14.3)
>5	336 (85.7)
**Education status**	
Secondary and above	33 (6.5)
Primary	191 (37.6)
Not educated	284 (55.9)
**Occupation status**	
Farmer	25 (4.9)
Day worker	50 (9.8)
Housewife	89 (17.5)
Not working	343 (67.5)
SES*	
High	156 (39.8)
Middle	80 (20.4)
Low	156 (39.8)
House’s structure*	
Compound of two rooms or more	204 (52)
Hut or one room	188 (48)
Source of water*	
Piped water	146 (37.2)
Others	246 (62.8)
Receiving ivermectin in the year preceding the study**	
Yes	217 (42.7)
No	291 (57.3)

SES, Socioeconomic status

*number of respondents was 392

**Ivermectin was not distributed in Al Marawi’ah and As Sukhnah districts.

### Seroprevalence of anti-*Ov*16 IgG4

The overall anti-*Ov*16 IgG4 seroprevalence rate was 18.5% (94/508), with a higher rate in Ad Dahi (23.7%) than Bani Sa’ad (20.4%), but there was no statistically significant difference (χ^2^ = 0.61, *P* = 0.435). On the other hand, lower rates of 8.0% and 6.1% were observed in Al Marawi'ah and As Sukhnah, respectively (Table 2).

**Table 2:** Age-stratified prevalence and distribution of anti-*Ov*16 IgG4 in Hodeidah and Al-Mahwit governorates of Tihama region, Yemen (2017).

District (sub-districts)	Prevalence of anti-*Ov*16 IgG4 stratified by age group (years)					
	≤10		>10		All ages	
	*n*/*N* (%)	95% CI	*n*/*N* (%)	95% CI	*n*/*N* (%)	95% CI
**Overall prevalence**	12/118 **(10.2)**	5.0–17.0	82/390 **(21.0)**	17.0–25.0	94/508 **(18.5)**	15.0–22.0
**Ivermectin-targeted districts endemic for onchocerciasis**						
**Bani Sa’ad**						
Al Wahaweh	1/17 (5.9)	0.2–29.0	16/54 (29.6)	18.0–44.0	17/71 (23.9)	15.0–36.0
Bani Ali	0/17 (0.0)	0.0–19.0	5/48 (10.4)	4.0–23.0	5/65 (7.7)	2.6–17.0
Gaaferat Alh	0/5 (0.0)	0.0–52.0	6/18 (33.3)	13.0–59.0	6/23 (26.1)	10.0–48.0
Utmah	4/16 (25)	7.0–52.0	10/31(32.3)	17.0–51.0	14/47 (29.8)	17.0–49.0
**Total**	5/55 **(9.1)**	3.0–20.0	37/151 **(24.5)**	18.0–32.0	42/206 **(20.4)**	15.0–27.0
**Ad Dahi**						
Upper Grabeh	1/4 (25)	0.6–81.0	15/52 (28.8)	17.0–43.0	16/56 (28.6)	17.0–42.0
Lower Grabeh	5/26 (19.2)	7.0–39.0	23/104 (22.1)	15.0–31.0	28/130 (21.5)	15.0–30.0
**Total**	6/30 **(20.4)**	7.0–52.0	38/156 **(24.4)**	19.0–32.0	44/186 **(23.7)**	18.0–30.0
**Ivermectin-untargeted districts with unknown onchocerciasis endemicity**						
**Al Marawi’ah**	1/5 **(20.0)**	0.5–72.0	3/45 **(6.7)**	1.4–18.0	4/50 **(8.0)**	2.2–19.0
**As Sukhnah**	0/28 **(0.0)**	0.0–12.0	4/38 **(10.5)**	2.9–25.0	4/66 **(6.1)**	1.7–15.0

*N*, Number of participants examined; *n*, number of anti-*Ov*16 IgG4-positive participants; CI, confidence interval.

### Age-stratified seroprevalence of anti-*Ov*16 IgG4

In Bani Sa’ad, the prevalence of anti-*Ov*16 IgG4 among participants aged ≤10 years was significantly lower (χ^2^ = 5.9, *P* = 0.015) than among those aged >10 years, being 9.1% and 24.5%, respectively. In contrast, no statistically significant difference (χ^2^ = 0.27, *P* = 0.610) was observed in the prevalence of anti-*Ov*16 IgG4 between the participants of the two age groups in Ad Dahi, being 20.4% and 24.4% for children aged ≤10 and >10 years, respectively. With the exception of anti-*Ov*16 IgG4 positivity in a seven-year-old participant from Al Marawi’ah, all participants tested positive for anti-*Ov*16 IgG4 in Al Marawi’ah and As Sukhnah were >10 years (Table 2).

### Factors associated with anti-*Ov*16 IgG4 seropositivity

Bivariate analysis showed that only the age and family size were significant predictors of anti-*Ov*16 IgG4 seropositivity, where those aged >10 years were at about a twice higher risk of exposure to *O*. *volvulus* infection than those aged ≤10 years (OR = 2.18; 95% CI: 1.10–4.31, *P* = 0.024). In addition, participants from large families were more than twice as likely to be exposed to infection compared to those from small families (OR = 2.6; 95% CI: 1.08–6.31, *P* = 0.028). The occupations of farmers (OR = 2.93; 95% CI: 1.25–6.88, *P* = 0.014) and housewives (OR = 2.15; 95% CI: 1.16–3.96, *P* = 0.015) were significantly associated with anti-Ov16 IgG4 seropositivity. However, district of residence (OR = 1.21; 95% CI: 0.75–1.95, *P* = 0.435), gender (OR = 1.03; 95% CI: 0.63–1.68, *P* = 0.914), education status (OR = 2.69; 95% CI: 0.78–9.27, *P* = 0.117), SES (OR = 1.0; 95% CI: 0.58–1.73; *P* = 1.00) source of water (OR = 1.00; 95% CI: 0.61–1.64; *P* = 0.994), history of ivermectin intake (OR = 1.01; 95% CI: 0.74–1.57; *P* = 0.693), presence of nodules and/or itching (OR = 1.06; 95% CI: 0.59–1.92; *P* = 0.839) was not found to be significantly associated with anti-Ov16 IgG4 seropositivity. On the other hand, multivariable analysis further confirmed that farmers (adjusted OR = 2.67; 95% CI: 1.11–6.44, *P* = 0.029), housewives (adjusted OR = 1.92; 95% CI: 1.01–3.64, *P* = 0.046) and being a member of a large family (adjusted OR = 2.62; 95% CI: 1.07–6.45, *P* = 0.063) were the independent risk factors associated with anti-*Ov*16 IgG4 seropositivity among residents of endemic rural areas of Hodeidah and Al-Mahwit (Table 3).

**Table 3:** Factors associated with anti-*Ov*16 IgG4 seropositivity among residents of onchocerciasis-endemic areas of Hodeidah and Al-Mahwit, Yemen (2017).

Variable	*N*	*n* (%)	OR (95% CI)	*P* value
**District of residence**				
Bani Sa’ad	206	42 (**20.4**)	Reference	
Ad Dahi	186	44 (**23.7**)	1.21 (0.75–1.95)	0.435
**Gender**				
Female	239	52 (**21.8**)	Reference	
Male	153	34 (**22.2**)	1.03 (0.63–1.68)	0.914
**Age** (years)				
≤10	85	11 (**12.9**)	Reference	
>10	307	75 (**24.4**)	2.18 (1.10–4.31)	0.024
**Family size** (members)				
≤5	56	6 (**10.7**)	Reference	
>5	336	80 (**23.8**)	2.6 (1.08–6.31)	0.028*
**Education status**				
Secondary and above	29	3 (**10.3**)	Reference	
Primary	152	33 (**21.7**)	2.40 (0.69–8.44)	0.171
Non-educated	211	50 (**23.7**)	2.69 (0.78–9.27)	0.117
**Occupation status**				
Not working	286	53 (**18.5**)	Reference	
Farmer	25	10 (**40.0**)	2.93 (1.25–6.88)	0.014*
Day worker	20	3 (**15.0**)	0.78 (0.22–2.74)	0.694
Housewife	61	20 (**32.8**)	2.15 (1.16–3.96)	0.015*
**SES**				
High	156	32 (**20.5**)	Reference	
Middle	80	22 (**27.5**)	1.5 (0.77–2.75)	0.228
Low	156	32 (**20.5**)	1.0 (0.58–1.73)	1.00
**Source of water**				
Piped water	146	32 (**21.9**)	Reference	
Others	246	54 (**22.0**)	1.0 (0.61–1.64)	0.994
**History of ivermectin intake**				
Yes	217	46 (**21.2**)	Reference	
No	175	40 (**22.9**)	1.01 (0.74–1.57)	0.693
Presence of nodules and/or skin itching**				
No	313	68 (**21.7**)	Reference	
Yes	79	18 (**22.8**)	1.06 (0.59–1.92)	0.839

*N*, Number of participants examined; *n*, number of anti-*Ov*16 IgG4-positive samples; OR, Odds ratio; CI, confidence interval; SES, socioeconomic status

* Confirmed as independent risk factors by multivariable analysis

** Nodules were observed with and without skin itching in 43 and two participants, respectively. The presence of skin itching only was reported by 34 participants.

## Discussion

Onchocerciasis is focally endemic in eight governorates of Yemen. Nevertheless, neither estimates of *O*. *volvulus* burden in the country nor studies on the impact of regular ivermectin campaigns or CDTI on its transmission in targeted areas are encountered published in the literature. Because of the failure to achieve the goal of eliminating the disease by 2015, the WHO paid attention to its elimination from the country by 2020 [33]. In Hodeidah and Al-Mahwit, control activities have been carried out by the CSSW since 2000, mainly through the distribution of ivermectin donated by the MDP to infected individuals. The last activity was the MDA to endemic districts in Hodeidah and Al-Mahwit through involving local populations in CDTI campaigns in 2016. To the best of our knowledge, the impact of campaigns in interrupting the transmission of the parasite in targeted areas has not been assessed in Yemen.

The present study revealed an anti-*Ov*16 IgG4 seroprevalence rate of 18.5% among local residents of the four study districts in Hodeidah and Al-Mahwit and seropositivity among young children, providing serologic evidence for ongoing *O*. *volvulus* transmission following regular ivermectin distribution to infected individuals and CDTI campaigns in such districts. This is in contrast to the success of ivermectin MDA campaigns to interrupt onchocerciasis transmission and its elimination from a number of countries and certain endemic foci in some countries of Africa and Latin America [10, 21, 22, 34–40]. However, it could be unrealistic to compare between the successful high-coverage efforts devoted by onchocerciasis elimination programs over a long time in such countries and the low-coverage campaigns implemented by an NGO in the governorates of Tihama. Moreover, the political upheaval and wars in the country since 2011 negatively impacted the efforts of disease elimination, rescheduling the expected disease elimination from the country by 2015 [17].

The present study is the first to unveil the transmission of the infection in districts with unknown disease endemicity, where prevalence rates of 8.0% and 6.1% were observed in the districts of Al Marawi'ah and As Sukhnah. This, in turn, indicates that the disease is probably more widespread than historically and anecdotally anticipated. However, infection needs to be confirmed by a skin-snip polymerase chain reaction (PCR). In addition, serologic assessment of infection among children according to the WHO guidelines is required to assess the ongoing transmission of onchocerciasis in such new foci. PCR screening of blackfly pools for the parasite could also augment post-MDA elimination mapping in areas alongside wadis and their tributaries. The lower anti-*Ov*16 IgG4 prevalence in the latter districts compared to Bani Sa’ad and Ad Dahi could be explained by the fact that endemicity levels of onchocerciasis vary between geographic areas as a result of the interaction between several factors related to the parasite, vector, host and environmental conditions. Thus, comprehensive mapping of endemic areas is needed to geostatistically determine the level of disease endemicity and the foci of top priority for targeting with ivermectin MDA campaigns. Meanwhile, the high proportion (21.7%) of asymptomatic infected patients in the present study reveals that symptom-free microfilaria carriers could be a potential reservoir of infection and contribute to the ongoing transmission of the parasite in such areas, particularly with the fact that they may deny the use of ivermectin in absence of symptoms. It is worth mentioning that asymptomatic *O*. *volvulus* infections are not uncommon events among Yemeni patients, possibly representing a third of cases in some endemic areas [11]. In contrast, hyper-reactive sowda patients have very low-density microfilariae in the skin and usually comply with ivermectin treatment. The low rate of nodule carriers in the studied districts (11.5%; 45/392) is consistent with the published literature about the uncommon presentation of subcutaneous nodules among Yemeni patients with sowda in earlier studies [15, 41, 42]. For instance, Anderson et al. [41] reported that 14.3% (5/35) of skin snip-positive patients from the southwestern region of Yemen were nodule carriers. In addition, Büttner and Racz [42] reported that only 15.0% (16/104) of patients with onchocerciasis in Taiz were nodule carriers, with nodules being mostly observed over the calf or thigh.

The lack of baseline anti-*Ov*16 IgG4 seroprevalence rates makes it difficult to accurately understand the extent to which ivermectin distribution had impacted the disease epidemiology. However, seropositivity rate among children ≤10 years in the studied districts confirms recent exposure and continuing transmission. It is noteworthy that anti-*Ov*16 IgG4 of <0.1% among children <10 years old is the criterion set by the WHO to confirm the interruption of disease transmission and its elimination [33, 43].

In the present study, the significantly lower rate among children aged ≤10 years compared to those >10 years (9.1% *vs*. 24.3%, respectively) in Bani Sa’ad raises promise regarding a partial impact of the ivermectin on onchocerciasis transmission. This could reflect the accumulative impact of the regular three-month-interval distribution of ivermectin to the affected individuals in Bani Sa’ad since 2000 prior to the last campaign in 2016. On the other hand, the single campaign in Ad Dahi in 2016 did not lead to significant changes in the prevalence of anti-*Ov*16 IgG4 between children aged ≤10 years (20.4%) and those >10 years (24.4%). The early start and repeated distribution of ivermectin in Bani Sa’ad could probably maintain drug coverage for the entire reproductive life span of *O*. *volvulus* adult worms, which may extend between 9 and 14 years [44]. It is to be noted that ivermectin is a long-acting microfilaricidal drug that has a little effect on the adult worms and, therefore, controls the disease by killing microfilariae, reducing clinical manifestations and interrupting transmission by the vector but does not cure the disease completely [45]. This, in turn, justifies for the rare exposure to *O*. *volvulus* among children born by the end of MDA implementation in endemic areas and the utility of screening such children for anti-*Ov*16 IgG4 as an indirect indicator for determining transmission interruption [33].

Mathematical modeling demonstrates the utility of anti-*Ov*16 IgG4 seropositivity among children aged <10 years as a marker for the post-MDA interruption of onchocerciasis transmission [46]. Yearly MDAs significantly reduce human infection rates and, hence, reduce seroconversion rates in newborns and young children proportionately to the MDA duration and coverage, but adults who seroconverted before the start of MDA remain seropositive [46]. Moreover, a lower force of infection (FOI) is usually associated with younger age groups [47], where FOI is an epidemiologic measure of the rate of infection acquisition by susceptible individuals and is usually used in mathematical modeling to compare the rate of disease transmission between two different groups [48].

Continuing transmission of *O*. *volvulus* after MDA with ivermectin has been suggested in three Senegalese districts after more than ten years of 45–90% coverage, where the anti-*Ov*16 IgG4 was prevalent among 6.9% of the population and 2.5% of five- to nine-year-old children [49]. On the other hand, the interruption of onchocerciasis transmission following years of MDA has been evidenced, among other criteria, by seronegativity or <0.1% seroprevalence of anti-*Ov*16 IgG4 among children aged ≤10 years in certain foci of endemic countries, including Guatemala [33, 34], Uganda [36], Mexico [37], Sudan [21, 38], Ecuador [22], Nigeria [39] and Equatorial Guinea [40].

In Yemen, skin snip examination for sowda is challenging due to the rare presence of microfilariae that may require repeated collection and examination of skin snips [14, 41]. This was evident in the present study, where all skin snips from nodule carriers were negative for microfilariae. This is attributed to both the poor sensitivity of skin snip microscopy for the detection of the low microfilarial load in the nodules of sowda patients due to their degeneration by the hyper-reactive immune response [42] and the possible impact of distributed ivermectin in killing microfilariae. As a rule of thumb, however, examination of skin snips should not be used to evaluate the impact of MDA with ivermectin on the interruption of onchocerciasis transmission or to determine the time of stopping such MDA campaigns [33].

In the present study, individuals aged >10 years are at a twice higher risk of exposure to *O*. *volvulus* infection compared to those aged ≤10 years. Repeated risk of contact with the vector could explain the higher exposure by increasing age. Moreover, the impact of ivermectin on reducing the infection exposure rate among young children and seroconversion of adults before the start of ivermectin administration could not be ruled out [46]. Although large family size was an independent risk factor of anti-*Ov*16 IgG4 positivity in the endemic districts, the reason behind this association is not clear but it could be attributed to the fact that more family members are engaged in agricultural activities and spend most daytime outside houses compared to small families with younger members. The significantly higher seropositivity rates among farmers and housewives compared to those not working could be explained by the increased exposure of these population categories to the bites of blackflies. In such rural communities, farmers and housewives are usually engaged in agricultural activities in the farms along the breeding sites of the vectors for long periods during the daytime. In addition, housewives are mainly responsible for bringing water from seasonal watercourses to their homes, usually several times a day based on the amount of the water needed by their household members. It is noteworthy that the vector of *O*. *volvulus* in Yemen belongs to *Simulium damnosum* complex, which is a distinctive subspecies referred to as *S*. *rasyani* and bites outdoors during the daytime with two peaks of biting activity, in the morning until 09.00 and after 16.00 [50, 51]. It should be stressed that there is a need for a detailed study of the factors affecting the extent of human contact with blackfly populations and the intensity of exposure to infection, including the distance of human dwellings from probable breeding sites.

Although the present study is limited by adopting only *Ov*16 serology as a criterion, its primary aim was to provide baseline seroprevalence of anti-*Ov*16 IgG4 in endemic areas of the country because of the lack of published studies in this regard. Moreover, skin snip microscopy is not suitable for the evaluation of transmission status and is absolutely insensitive for the detection of microfilariae in case of sowda. On the other hand, it was rather difficult to meet the entomologic criterion set by the WHO [33], which recommends a minimum sample size of 6000 blackflies to determine the prevalence of infective flies by PCR. In fact, this is in accordance with the recommendations of the WHO Guideline Development Group [33], which declared that resources, cost, feasibility and acceptability should be considered when choosing the tests to be used to demonstrate the interruption of onchocerciasis transmission.

It has to be acknowledged is that there is no prior validation of RDTs in the study area against *Ov*16 ELISA as a reference method, and this comes in part from the unavailability of commercial ELISA kits for this purpose. Nevertheless, the quality of RDT performance has been assured by the inclusion of serial dilutions of anti-*Ov*16 IgG4 positive and negative controls supplied by PATH (www.path.org). Moreover, the feasibility of integrating the use of anti-*Ov*16 IgG4 RDTs into onchocerciasis surveillance activities instead of the use of traditional skin snip microscopy has been recently demonstrated from Senegal [52]. Another issue to be considered is that the numbers of investigated cases are few when allocated over the four districts of the study. However, this could be justified by the fact the total sample size was statistically calculated to address the study objective.

In conclusion, onchocerciasis is still being transmitted in the Tihama region of Yemen despite ivermectin distribution to the affected individuals and the implementation of CDTI in 2016. This is supported by the recent exposure of children aged ≤10 years in the region to the parasite, who were positive for anti-*Ov*16 IgG4. This could be attributed to the insufficient coverage rate with the drug and its distribution without having baseline infection rates in the targeted endemic areas and their neighboring localities. Moreover, onchocerciasis transmission has also been found in two districts not previously categorized as endemic for the disease and had never been targeted by ivermectin. Therefore, there is a need to establish valid baseline data for onchocerciasis mapping in endemic or potentially endemic areas before being targeted by ivermectin MDAs, with continuous monitoring with respect to elimination mapping. Despite the absence of onchocerciasis interruption, there is a decline in disease transmission in Bani Sa’ad district of Al-Mahwit as reflected by the significantly lower anti-*Ov*16 IgG4 seroprevalence among children aged ≤10 years. Therefore, interruption of disease transmission and its elimination is most likely a future task if good coverage with regular ivermectin MDA campaigns is achieved and its impact on disease transmission is continually monitored and evaluated.

## Supporting information

S1 Checklist STROBE checklist. (DOC)

## References

[1] World Health Organization. Onchocerciasis. Fact sheet N°374: World Health Organization; 2015 [updated March 2015; cited 2015 29 Jan.]. Available from: http://www.who.int/mediacentre/factsheets/fs374/en/.

[2] World Health Organization. Progress report on the elimination of human onchocerciasis, 2015–2016. Wkly Epidemiol Rec. 2016; 91(43): 505–14. 27801998

[3] Global Burden of Disease 2015 DALYs, HALE Collaborators. Global, regional, and national disability-adjusted life-years (DALYs) for 315 diseases and injuries and healthy life expectancy (HALE), 1990–2015: a systematic analysis for the Global Burden of Disease Study 2015. Lancet 2016; 388(10053):1603–58. doi: 10.1016/S0140-6736(16)31460-X 27733283 10.1016/S0140-6736(16)31460-X PMC5388857

[4] Gaxotte P. Onchocerciasis and the Mectizan Donation Program. Sante 1998; 8(1):9–11. 9592868

[5] Peters DH, Phillips T. Mectizan Donation Program: evaluation of a public-private partnership. Trop Med Int Health 2004; 9(4):A4–15. doi: 10.1111/j.1365-3156.2004.01209.x 15078275 10.1111/j.1365-3156.2004.01209.x

[6] Alleman MM, Twum-Danso NA, Thylefors BI. The Mectizan Donation Program—highlights from 2005. Filaria J 2006; 5:11 doi: 10.1186/1475-2883-5-11 17005039 10.1186/1475-2883-5-11 PMC1618829

[7] Colatrella B. The Mectizan Donation Program: 20 years of successful collaboration—a retrospective. Ann Trop Med Parasitol 2008; 102 Suppl 1:7–11. doi: 10.1179/136485908X337418 18718147 10.1179/136485908X337418

[8] Diawara L, Traore MO, Badji A, Bissan Y, Doumbia K, Goita SF, et al Feasibility of onchocerciasis elimination with ivermectin treatment in endemic foci in Africa: first evidence from studies in Mali and Senegal. PLoS Negl Trop Dis. 2009; 3(7):e497 doi: 10.1371/journal.pntd.0000497 19621091 10.1371/journal.pntd.0000497 PMC2710500

[9] Tekle AH, Elhassan E, Isiyaku S, Amazigo UV, Bush S, Noma M, et al Impact of long-term treatment of onchocerciasis with ivermectin in Kaduna State, Nigeria: first evidence of the potential for elimination in the operational area of the African Programme for Onchocerciasis Control. Parasit Vectors 2012; 5:28 doi: 10.1186/1756-3305-5-28 22313631 10.1186/1756-3305-5-28 PMC3296569

[10] World Health Organization. Progress towards eliminating onchocerciasis in the WHO Region of the Americas: verification of elimination of transmission in Guatemala. Wkly Epidemiol Rec 2016; 91(43):501–5. 27801556

[11] Büttner DW, von Laer G, Mannweiler E, Büttner M. Clinical, parasitological and serological studies on onchocerciasis in the Yemen Arab Republic. Tropenmed Parasitol. 1982; 33(4):201–12. 7164162

[12] Abdul-Ghani R, Mahdy MA, Beier JC. Onchocerciasis in Yemen: Time to take action against a neglected tropical parasitic disease. Acta Trop. 2016; 162:133–41. doi: 10.1016/j.actatropica.2016.06.017 27325293 10.1016/j.actatropica.2016.06.017

[13] Richard-Lenoble D, Al Qubati Y, Toe L, Pisella PJ, Gaxotte P, al Kohlani A. Human onchocerciasis and "sowda" in the Republic of Yemen. Bull Acad Natl Med. 2001; 185(8):1447–59. 11974966

[14] Connor DH, Gibson DW, Neafie RC, Merighi B, Buck AA. Sowda—onchocerciasis in north Yemen: a clinicopathologic study of 18 patients. Am J Trop Med Hyg. 1983; 32(1):123–37. 6824118 10.4269/ajtmh.1983.32.123

[15] Omar MS, Franz M, Büttner DW. Some observations on onchocerciasis including sowda in the Yemen Arab Republic. Tropenmed Parasitol. 1979; 30(1):113–9. 442197

[16] Al-Qubati Y. The first use of ivermectin for the treatment of onchocerciasis in Yemen. Trans R Soc Trop Med Hyg. 1994; 88(3):343 7974684 10.1016/0035-9203(94)90109-0

[17] World Health Organization. Sustaining the drive to overcome the global impact of neglected tropical diseases—second WHO report on neglected tropical diseases WHO/HTM/NTD/2013.1 Geneva: WHO; 2013 [cited 2017 24 July]. Available from: http://www.who.int/iris/bitstream/10665/77950/1/9789241564540_eng.pdf.

[18] World Health Organization. Neglected tropical diseases: Fighting NTDs in Yemen [cited 2018 15 January]. Available from: http://www.who.int/neglected_diseases/yemen/en/.

[19] Lobos E, Weiss N, Karam M, Taylor HR, Ottesen EA, Nutman TB. An immunogenic *Onchocerca volvulus* antigen: a specific and early marker of infection. Science 1991; 251(5001):1603–5. 2011741 10.1126/science.2011741

[20] Lakwo TL, Garms R, Rubaale T, Katabarwa M, Walsh F, Habomugisha P, et al The disappearance of onchocerciasis from the Itwara focus, western Uganda after elimination of the vector *Simulium neavei* and 19 years of annual ivermectin treatments. Acta Trop. 2013;126(3):218–21. doi: 10.1016/j.actatropica.2013.02.016 23458325 10.1016/j.actatropica.2013.02.016

[21] Higazi TB, Zarroug IM, Mohamed HA, Elmubark WA, Deran TC, Aziz N, et al Interruption of *Onchocerca volvulus* transmission in the Abu Hamed focus, Sudan. Am J Trop Med Hyg. 2013;89(1):51–7. doi: 10.4269/ajtmh.13-0112 23690554 10.4269/ajtmh.13-0112 PMC3748488

[22] Lovato R, Guevara A, Guderian R, Proano R, Unnasch T, Criollo H, et al Interruption of infection transmission in the onchocerciasis focus of Ecuador leading to the cessation of ivermectin distribution. PLoS Negl Trop Dis. 2014; 8(5):e2821 doi: 10.1371/journal.pntd.0002821 24853587 10.1371/journal.pntd.0002821 PMC4031166

[23] Convit J, Schuler H, Borges R, Olivero V, Dominguez-Vazquez A, Frontado H, et al Interruption of *Onchocerca volvulus* transmission in Northern Venezuela. Parasit Vectors 2013; 6(1):289 doi: 10.1186/1756-3305-6-289 24499653 10.1186/1756-3305-6-289 PMC3856516

[24] Oguttu D, Byamukama E, Katholi CR, Habomugisha P, Nahabwe C, Ngabirano M, et al Serosurveillance to monitor onchocerciasis elimination: the Ugandan experience. Am J Trop Med Hyg. 2014;90(2):339–45. doi: 10.4269/ajtmh.13-0546 24343885 10.4269/ajtmh.13-0546 PMC3919245

[25] Golden A, Steel C, Yokobe L, Jackson E, Barney R, Kubofcik J, et al Extended result reading window in lateral flow tests detecting exposure to *Onchocerca volvulus*: a new technology to improve epidemiological surveillance tools. PLoS One 2013; 8(7):e69231 doi: 10.1371/journal.pone.0069231 23935960 10.1371/journal.pone.0069231 PMC3720650

[26] Steel C, Golden A, Stevens E, Yokobe L, Domingo GJ, de los Santos T, et al Rapid point-of-contact tool for mapping and integrated surveillance of *Wuchereria bancrofti* and *Onchocerca volvulus* Infection. Clin Vaccine Immunol. 2015; 22(8):896–901. doi: 10.1128/CVI.00227-15 26018537 10.1128/CVI.00227-15 PMC4519720

[27] Solomon AW, Engels D, Bailey RL, Blake IM, Brooker S, Chen JX, et al A diagnostics platform for the integrated mapping, monitoring, and surveillance of neglected tropical diseases: rationale and target product profiles. PLoS Negl Trop Dis. 2012; 6(7):e1746 doi: 10.1371/journal.pntd.0001746 22860146 10.1371/journal.pntd.0001746 PMC3409112

[28] WHO African Programme for Onchocerciasis Control. Report of the fortieth session of the Technical Consultative Committee (TCC) DIR/PM/APOC/REP/TCC40. Ouagadougou: WHO/APOC; 2015.

[29] Golden A, Stevens EJ, Yokobe L, Faulx D, Kalnoky M, Peck R, et al A recombinant positive control for serology diagnostic tests supporting elimination of *Onchocerca volvulus*. PLoS Negl Trop Dis 2016; 10(1):e0004292 doi: 10.1371/journal.pntd.0004292 26745374 10.1371/journal.pntd.0004292 PMC4706346

[30] Lwanga SK, Lemeshow S. Sample size determination in health studies: a practical manual Geneva: World Health Organization; 1991.

[31] World Health Organization. Onchocerciasis and its control. Report of a WHO Expert Committee on Onchocerciasis Control. World Health Organ Tech Rep Ser 1995;852:1–104. 7541171

[32] Vyas S, Kumaranayake L. Constructing socio-economic status indices: how to use principal components analysis. Health Policy Plan 2006;21(6):459–68. doi: 10.1093/heapol/czl029 17030551 10.1093/heapol/czl029

[33] World Health Organization. Guidelines for stopping mass drug administration and verifying elimination of human onchocerciasis: criteria and procedures Geneva: WHO, 2016 WHO/HTM/NTD/PCT/2016.1. 26913317

[34] Lindblade KA, Arana B, Zea-Flores G, Rizzo N, Porter CH, Dominguez A, et al Elimination of *Onchocercia volvulus* transmission in the Santa Rosa focus of Guatemala. Am J Trop Med Hyg. 2007;77(2):334–41. 17690408

[35] Richards F Jr., Rizzo N, Diaz Espinoza CE, Monroy ZM, Crovella Valdez CG, de Cabrera RM, et al One hundred years after its discovery in Guatemala by Rodolfo Robles, *Onchocerca volvulus* transmission has been eliminated from the Central Endemic Zone. Am J Trop Med Hyg. 2015;93(6):1295–304. doi: 10.4269/ajtmh.15-0364 26503275 10.4269/ajtmh.15-0364 PMC4674249

[36] Katabarwa MN, Walsh F, Habomugisha P, Lakwo TL, Agunyo S, Oguttu DW, et al Transmission of onchocerciasis in Wadelai focus of northwestern Uganda has been interrupted and the disease eliminated. J Parasitol Res. 2012; 2012:748540 doi: 10.1155/2012/748540 22970347 10.1155/2012/748540 PMC3433138

[37] Rodriguez-Perez MA, Dominguez-Vazquez A, Unnasch TR, Hassan HK, Arredondo-Jimenez JI, Orozco-Algarra ME, et al Interruption of transmission of *Onchocerca volvulus* in the Southern Chiapas Focus, Mexico. PLoS Negl Trop Dis 2013; 7(3):e2133 doi: 10.1371/journal.pntd.0002133 23556018 10.1371/journal.pntd.0002133 PMC3610615

[38] Zarroug IM, Hashim K, ElMubark WA, Shumo ZA, Salih KA, ElNojomi NA, et al The first confirmed elimination of an onchocerciasis focus in Africa: Abu Hamed, Sudan. Am J Trop Med Hyg. 2016;95(5): 1037–40. doi: 10.4269/ajtmh.16-0274 27352878 10.4269/ajtmh.16-0274 PMC5094213

[39] Evans DS, Alphonsus K, Umaru J, Eigege A, Miri E, Mafuyai H, et al Status of onchocerciasis transmission after more than a decade of mass drug administration for onchocerciasis and lymphatic filariasis elimination in central Nigeria: challenges in coordinating the stop MDA decision. PLoS Negl Trop Dis. 2014; 8(9):e3113 doi: 10.1371/journal.pntd.0003113 25233351 10.1371/journal.pntd.0003113 PMC4169246

[40] Moya L, Herrador Z, Ta-Tang TH, Rubio JM, Perteguer MJ, Hernandez-Gonzalez A, et al Evidence for suppression of onchocerciasis transmission in Bioko Island, Equatorial Guinea. PLoS Negl Trop Dis. 2016; 10(7):e0004829 doi: 10.1371/journal.pntd.0004829 27448085 10.1371/journal.pntd.0004829 PMC4957785

[41] Anderson J, Fuglsang H, al-Zubaidy A. Onchocerciasis in Yemen with special reference to sowda. Trans R Soc Trop Med Hyg. 1973; 67(1):30–1. 4777417 10.1016/0035-9203(73)90300-3

[42] Büttner DW, Racz P. Macro- and microfilariae in nodules from onchocerciasis patients in the Yemen Arab Republic. Tropenmed Parasitol 1983; 34(2):113–21. 6879705

[43] World Health Organization. Certification of elimination of human onchocerciasis criteria and procedures Geneva: WHO, 2001 WHO/CDS/CPE/CEE/2001.18b.

[44] Plaisier AP, van Oortmarssen GJ, Remme J, Habbema JD. The reproductive lifespan of *Onchocerca volvulus* in West African savanna. Acta Trop 1991; 48(4):271–84. 1674401 10.1016/0001-706x(91)90015-c

[45] Ette EI, Thomas WO, Achumba JI. Ivermectin: a long-acting microfilaricidal agent. DICP 1990; 24(4):426–33. 2183496 10.1177/106002809002400417

[46] Lont YL, Coffeng LE, de Vlas SJ, Golden A, de Los Santos T, Domingo GJ, et al Modelling anti-*Ov*16 IgG4 antibody prevalence as an indicator for evaluation and decision making in onchocerciasis elimination programmes. PLoS Negl Trop Dis. 2017; 11(1):e0005314 doi: 10.1371/journal.pntd.0005314 28114304 10.1371/journal.pntd.0005314 PMC5289624

[47] Golden A, Faulx D, Kalnoky M, Stevens E, Yokobe L, Peck R, et al Analysis of age-dependent trends in *Ov*16 IgG4 seroprevalence to onchocerciasis. Parasit Vectors 2016; 9(1):338 doi: 10.1186/s13071-016-1623-1 27296630 10.1186/s13071-016-1623-1 PMC4907250

[48] Mueller I, Schoepflin S, Smith TA, Benton KL, Bretscher MT, Lin E, et al Force of infection is key to understanding the epidemiology of *Plasmodium falciparum* malaria in Papua New Guinean children. Proc Natl Acad Sci U S A. 2012; 109(25):10030–5. doi: 10.1073/pnas.1200841109 22665809 10.1073/pnas.1200841109 PMC3382533

[49] Wilson NO, Badara Ly A, Cama VA, Cantey PT, Cohn D, Diawara L, et al Evaluation of lymphatic filariasis and onchocerciasis in three Senegalese districts treated for onchocerciasis with ivermectin. PLoS Negl Trop Dis. 2016; 10(12):e0005198 doi: 10.1371/journal.pntd.0005198 27926918 10.1371/journal.pntd.0005198 PMC5142766

[50] Garms R, Kerner M. Anthropophily of *Simulium damnosum* s.l. and its role as a vector of human onchocerciasis in the Yemen Arab Republic. Tropenmed Parasitol. 1982; 33(3):175–80. 7135475

[51] Garms R, Kerner M, Meredith SE. *Simulium* (*Edwardsellum*) *rasyani* n.sp., the Yemen species of the *Simulium damnosum* complex. Trop Med Parasitol. 1988; 39(3):239–44 3194668

[52] Dieye Y, Storey HL, Barrett KL, Gerth-Guyette E, Di Giorgio L, Golden A, et al Feasibility of utilizing the SD BIOLINE Onchocerciasis IgG4 rapid test in onchocerciasis surveillance in Senegal. PLoS Negl Trop Dis. 2017; 11(10):e0005884 doi: 10.1371/journal.pntd.0005884 28972982 10.1371/journal.pntd.0005884 PMC5640270

